# Automating the overburdened clinical coding system: challenges and next steps

**DOI:** 10.1038/s41746-023-00768-0

**Published:** 2023-02-03

**Authors:** Kaushik P. Venkatesh, Marium M. Raza, Joseph C. Kvedar

**Affiliations:** Harvard Medical School, Boston, MA USA

**Keywords:** Health services, Health care economics

## Abstract

Artificial intelligence (AI) and natural language processing (NLP) have found a highly promising application in automated clinical coding (ACC), an innovation that will have profound impacts on the clinical coding industry, billing and revenue management, and potentially clinical care itself. Dong et al. recently analyzed the technical challenges of ACC and proposed future directions. Primary challenges for ACC exist at the technological and implementation levels; clinical documents are redundant and complex, code sets like the ICD-10 are rapidly evolving, training sets are not comprehensive of codes, and ACC models have yet to fully capture the logic and rules of coding decisions. Next steps include interdisciplinary collaboration with clinical coders, accessibility and transparency of datasets, and tailoring models to specific use cases.

Artificial intelligence (AI) and natural language processing (NLP) have found a highly promising application in automated clinical coding (ACC), an innovation that will have profound impacts on the clinical coding industry, billing and revenue management, and potentially clinical care itself. Clinical coding involves the systematic classification of medical records for billing as well as the tracking of clinical care data over time^1^. Every patient-provider interaction can be broken down into services and medical goods provided to the patient, which are captured across the electronic health record (EHR) in discrete data points (e.g. specific diagnoses) and unstructured free text medical notes.

In the US, one of the predominant coding classification systems is the ICD-10-CM (International Classification of Diseases, Tenth Revision, Clinical Modification), containing around 68,000 diagnosis codes^2^. Other important coding systems include CPT codes and HCPCS codes for health care services. Within these different code systems, every single diagnosis made and service provided is categorized for the purpose of record keeping and billing.

Clinical coders perform the resource-intensive process of manual coding, which involves textual analysis, summarization, and code classification. Coders require months of training and can code around 60 cases per day. Even at this rate, cases pending coding can be backlogged by months^3^. Moreover, the manual coding process is prone to errors—accuracy ranges widely (50–98%; median of 80%) depending on the coder, diagnosis/service, patient complexity, etc^4,5^.

Given the language-based, pattern-heavy, data-driven nature of coding decisions, AI and NLP offer the promise of ACC to support coders. Dong et al. recently analyzed the technical challenges of ACC and proposed future directions^3^. They also discuss the most accurate ACC system to date, which used a benchmark dataset (MIMIC-III) of US intensive care documents and ICD-9 codes^6^.

## Challenges with ACC

The first challenge Dong et al. identify is the varied structure, quality, and length of clinical documents used in coding. Clinical documents come in various forms, including discharge summaries, radiology reports, and auxiliary health professional notes. For reference, the average length of an intensive care discharge summary was 1500 words in the MIMIC-III dataset^7^. Much of the data in clinical documents is redundant. This includes “Note Bloat”, the common copied-and-pasted information in clinician notes that has been shown to affect the predictive ability of ACC models^8^. De-duplication of Note Bloat is one such way to process the superfluous data in clinical documents.

Additionally, many codes present in coding systems like ICD-10 are unlikely to appear more than a handful of times within a training datasets. For example, in the MIMIC-III dataset (using the 8932 codes in the now-outdated ICD-9), 5000 codes appeared less than 10 times and more than half of the codes never appeared at all^9^. These codes are considered “few-shot” and “zero-shot” learning problems. Integrating the logical rules of clinical coding into ACC models may help improve the chance of appropriately addressing these cases^10^.

Integrating the logic of coding guidelines into pattern-based ACC models is another challenge. Clinical coders are often required to synthesize data across different sources, some of which may be contradictory or irrelevant to final coding decisions. Deep learning AI models are trained on associations between data and codes, rather than algorithmic thinking—a threat. to accurate and reproducible ACC coding decisions. Integrating coding guideline logic into the ACC model is necessary to go beyond the typical “black-box” pattern-based AI^11^. One such study was able to formalize and integrate coding rules into an early ACC model^12^.

Finally, even once a system is trained with the logic of a particular code set like ICD-10, there will certainly be revisions to the code catalogs (ICD-11 was released in 2022^13^). Existing ACC models may potentially become inapplicable as ICD-11 and other updated code sets are implemented. Transitions of code sets could require new methods of data handling and mapping^14,15^.

## Next steps for ACC

The US clinical coding market was valued at $18 billion in 2021, and is expected to grow 8.0% annually until 2030^16^. This sizable market has stimulated the race to create the first widely adopted ACC model. Several large technology companies have already created semi-ACC systems, including Deloitte, Optum, and Capita^3^. Start-up AKASA recently created an ACC solution that outperformed human coders on the MIMIC-III dataset^17^. As more innovators and models enter the space, there remain three key next steps for the future of ACC.

The first is interdisciplinary collaboration; clinical coders must be involved in both the development and refinement of ACC models. Corrections, highlights, and new rules identified by human coders are essential forms of feedback that should be integrated into ACC algorithms. ACC software should include an interface for coders to provide this feedback, as some innovators have already done^18,19^.

A second important direction is accessibility and transparency. To support continued research and development, gold standard datasets from more health systems should be made publicly available. These datasets should be coded by experienced coders and validated according to standardized guidelines. Examples include the r-TERIFIC and BioNLP datasets^20,21^. Transparency is key considering that the outcomes of ACC decisions will affect billing and potentially clinical care decisions. In the pursuit of transparent billing behavior and contract negotiations, logic, data quality, and predictive validity of ACC models should be easily auditable^22^.

Third, ACC systems can also serve different needs depending on the types of codes they are designed to produce, e.g., billing and research. Billing requires broad-stroke codes to predict Diagnosis-Related Groups (DRGs) that determine fee-for-service billing. New payment models like capitated/global budget payments may require higher granularity of codes to track process and outcomes measures. Research also requires a high degree of granularity - case detection, phenotyping, and other aspects of research are more productive when able to use the full gamut of codes. Customized research-specific coding can also be implemented on top of existing ACC systems^23^.

## Conclusion

Altogether, there remain several innovation and adoption milestones yet to be reached by ACC technology. With collaboration between coders and developers, increased data for training, and continued progress in AI and NLP, we will surely see more advances in ACC in the coming years. The adoption and integration of ACC models, both assistive and autonomous, will have important ramifications for the coding industry as well as revenue management and billing for payers and providers.
